# Respectful maternal and newborn care: building a common agenda

**DOI:** 10.1186/s12978-015-0042-7

**Published:** 2015-05-20

**Authors:** Emma Sacks, Mary V. Kinney

**Affiliations:** Department of International Health, Johns Hopkins School of Public Health, 615 N. Wolfe St, E8011, Baltimore, MD 21205 USA; USAID Maternal and Child Survival Program (MCSP)/ICF International, Washington, DC USA; Save the Children, Saving Newborn Lives, 102 Louis Thibault Drive, Edgemead, 7441 South Africa

**Keywords:** Newborn health, Neonatal survival, Maternal health, Stillbirth, Preterm birth, Respectful care, Global agenda, Post-2015

## Abstract

In September, the World Health Organization released a statement on preventing and eliminating disrespect and abuse during facility-based childbirth. In addition to this important agenda, attention is also needed for the dignified care of newborns, who also deserve basic human rights and dignified care. In this commentary, we provide examples from the literature and other sources of where respectful care for newborns has been lacking and we give examples of opportunities for integration of maternal and newborn health care going forward. We illustrate the need for respectful treatment and consideration across the continuum of care: for mothers, stillbirths, and all newborns, including those born too soon and those who die in infancy. We explain the need to document cases of neglect and abuse, count all births and deaths, and to include newborns and stillbirths in the respectful care agenda and the post-2015 global reproductive care frameworks.

## Respectful maternal and newborn care: Building a common agenda

In September 2014, the World Health Organization released a statement on preventing and eliminating disrespect and abuse during facility-based childbirth [1]. This statement is a critical step for improving the reproductive care of women. It rightfully acknowledges that while “disrespectful and abusive treatment of women may occur throughout pregnancy, childbirth and the postpartum period, women are particularly vulnerable during childbirth” [1]. Babies are likewise vulnerable to neglect and disrespect during this time period; however, newborns are not mentioned in this statement except when women and infants are detained at facilities for inability to pay for services. Disregard for the needs of the family in cases of stillbirth^1^ or intra-partum death is not considered. Poor treatment and neglect of the infant outright are glaringly absent [1].

The WHO statement argues that “every woman has the right to the highest attainable standard of health, which includes the right to dignified, respectful health care” [1]. This standard must also be upheld for newborns and stillborn babies, yet anecdotal evidence suggests that various abuses occur. There have been reports of newborns left unattended, unnecessarily separated from their mothers after birth, and transferred to other facilities without consent of the parents [2]. Other claims have included unsafe early discharge of women and their infants due to limited space, discrimination against infants with congenital anomalies or illnesses and refusal of postnatal care for inability to pay [2-4]. Many of the 2.9 million neonatal deaths per year could be averted with timely and skilled intrapartum and neonatal care [5]; improved access to facility delivery is a cornerstone of improving maternal and neonatal survival and preventing stillbirths. Yet patients' experiences or expectations of abuse and disrespect act as a disincentive to seek necessary care.

It has been argued that “children should be viewed as having the right to be breastfed, not in the sense that the mother is obligated to breastfeed the child, but in the sense that no one may interfere with the mother's right to breastfeed the child” [6]; thus, facilitation of early initiation of breastfeeding constitutes an important practice of protecting maternal and newborn care in concert. Relatedly, stigma against both mothers and newborns who are HIV-exposed or infected persists as a serious challenge, both to the reduction of paediatric HIV infection and to overall neonatal care [7]. In the most extreme cases, there has even been documented evidence of female infanticide in parts of Asia [8] and twin infanticide in parts of south-western Nigeria [9], but even this has not been elevated to the global health agenda.

Babies born preterm^2^ or with other perinatal complications are at increased mortality risk in the first hours and days of life, and deserve to be given as much chance to live as their full term counterparts [10]. In places where fatalism on the part of health care workers is common [4], a culture of capability should be promoted. This requires governments and facilities to prioritize training, equipment and sufficient skilled personnel to handle both a new mother and a fragile, potentially critically-ill newborn, with competence and patience, at any given time; yet many facilities find a single nurse attending to multiple deliveries [2]. Infants born with terminal or severe chronic illness may require palliative care, yet analgesic medications, skilled administrators and culturally-competent counsellors are both in limited supply and inequitably distributed in most low-resource settings [11]. Comprehensive and sensitive care for mothers and newborns should recognize and support the individual needs and choices of each family beyond childbirth and into the post-partum period.

Furthermore, respectful care should not end with death; dignified care matters to grieving parents and communities. Respectful maternal and newborn care should include the option for mothers to acknowledge or hold a baby that has died, yet the bodies of stillborn babies are often disposed of without any recognition such as being named, dressed or given a funeral [12]. Most of the 2.6 million stillbirths that occur globally every year are never recorded and both stillbirths and intra-partum deaths continue to be miscategorised and undercounted [5]. Comprehensive health care extends beyond clinical care: stillbirths and early neonatal deaths should have both vital events properly documented, and death reviews should be conducted when warranted, to guide quality improvements where death and disability could have been avoided. In cases where women feel that they or their babies have been abused, mistreated or neglected, there are few avenues for legal recourse [13].

The need to document abusive and neglectful care, of all levels of severity, is paramount in efforts to reduce abuses and improve care of mothers during childbirth. Attempts to define disrespect and abuse of women in childbirth will be strengthened and made more accurate by broadening the scope to include newborns and stillbirths. Lynn Freedman and colleagues have emphasised the importance of high-quality care for women as an essential aspect of respectful maternity care [14]; this should extend to neonatal care, which is often provided at the same time and by the same health worker. In *The Lancet* Every Newborn series, Ann Starrs argues, “the maternal and newborn health communities need to pledge to each other that any policy, programme or initiative focusing on either maternal or newborn health will incorporate the other as well” [15]; this holds true for statements on disrespect and abuse. With clear evidence supporting access to high-quality, skilled care for all women [16], the concept of the mother-baby dyad must be prominent [15]. Improving quality of care, including respectful care, around the time of birth provides a triple return on investment, saving mothers and newborns and reducing the risk of stillbirths and disability [5].

As experts gather in the next few months to discuss the post-Millennium Development Goal era, the agenda on respectful care must be central and focused on both women and their babies. Global initiatives such as the Every Newborn Action Plan [17] and Ending Preventable Maternal Mortality [18] should inform this effort, ensuring the right to go through pregnancy, birth and the first month of life with the expectation to live, be counted and thrive [5]. Addressing disrespect and abuse in childbirth for mother and baby could move “integration” from a buzz word to actual practice; the upcoming Global Maternal and Newborn Health Conference in Mexico City in October 2015 is one step in harmonizing our efforts towards this goal. Global frameworks, including the Sustainable Development Goals, are another; we need to prioritize activities that monitor and prevent stillbirths, intra-partum and neonatal deaths, and fund and implement strategies to humanize our care of all patients and family members across the reproductive health continuum.

As we work to eliminate the preventable maternal and newborn deaths and stillbirths, we should do so in a conscientious manner. Beyond survival and basic health care, we must strive for in a conscientious manner respectful care for women and their babies together.

## Endnotes

^1^Stillbirth refers to all pregnancy losses after 22 weeks of gestation, but for numerical comparisons between international data, The Lancet Stillbirth group uses the WHO definition of a birth weight of at least 1000 g or a gestational age of at least 28 weeks (third-trimester stillbirth).

^2^Preterm birth is defined as a live birth prior to the completion of 37 weeks of pregnancy. This includes moderate to late preterm (32 to <37 weeks), very preterm (28 to <32 weeks), and extremely preterm (<28 weeks).
